# Signals and Their Perception for Remodelling, Adjustment and Repair of the Plant Cell Wall

**DOI:** 10.3390/ijms24087417

**Published:** 2023-04-18

**Authors:** Ralf Oelmüller, Yu-Heng Tseng, Akanksha Gandhi

**Affiliations:** Matthias Schleiden Institute of Genetics, Bioinformatics and Molecular Botany, Department of Plant Physiology, Friedrich-Schiller-University, 07743 Jena, Germany

**Keywords:** cell wall, cell wall integrity, malectin-domain containing proteins, cellulose, hemicelluloses, pectin, pattern recognition receptors, Arabidopsis

## Abstract

The integrity of the cell wall is important for plant cells. Mechanical or chemical distortions, tension, pH changes in the apoplast, disturbance of the ion homeostasis, leakage of cell compounds into the apoplastic space or breakdown of cell wall polysaccharides activate cellular responses which often occur via plasma membrane-localized receptors. Breakdown products of the cell wall polysaccharides function as damage-associated molecular patterns and derive from cellulose (cello-oligomers), hemicelluloses (mainly xyloglucans and mixed-linkage glucans as well as glucuronoarabinoglucans in Poaceae) and pectins (oligogalacturonides). In addition, several types of channels participate in mechanosensing and convert physical into chemical signals. To establish a proper response, the cell has to integrate information about apoplastic alterations and disturbance of its wall with cell-internal programs which require modifications in the wall architecture due to growth, differentiation or cell division. We summarize recent progress in pattern recognition receptors for plant-derived oligosaccharides, with a focus on malectin domain-containing receptor kinases and their crosstalk with other perception systems and intracellular signaling events.

## 1. Introduction

The cell wall defines the shape of a plant cell and the habitus of the organs of a plant. It is essential for the integrity of the cellular structure and resists internal hydrostatic pressure; regulates cell, and consequently, plant growth; provides sufficient flexibility to support cell division and expansion, acts as an environmental barrier against biotic and abiotic stresses and provides a milieu that allows cell-to-cell communication. The wall is important for the cell fate, its differentiation and many developmental processes. The pressure from the surrounding environment, in particular microorganisms, which use the wall material as an energy source, shapes the cell wall structure and potentially drove its evolution (cf. [1,2] and ref. therein). Therefore, monitoring the status and integrity of the cell wall is important for a cell, and responses to cell wall alterations must combine information ranging from cell-internal developmental programs to severe damage by external threats.

Breakdown products of the cell wall polymers function as sensors or damage-associated molecular patterns (DAMPs) which are recognized by apoplastic proteins and apoplastic epitopes of cell surface receptors. We will first describe the cell wall polysaccharides, their synthesis and degradation to elicitor-active oligosaccharides. Moreover, tension and altered ion gradients across the plasma membrane are perceived by the cell and coordinated with information from accumulating oligosaccharides. Furthermore, cell wall damage occurs during abiotic or biotic threat. Information about the kind of threat further fine-tunes the response of the cell. Therefore, crosstalk between oligosaccharide perception/signaling and signals from other DAMPs, microbe/pathogen-associated molecular patterns (M/PAMPs) or cell-internal changes is important to establish an appropriate cellular response, which occurs during signal perception and/or signaling. We summarize recent progress and novel players involved in the perception of cell wall breakdown products, their potential interactions and crosstalk to other perception systems and induced signaling pathways.

## 2. Structure and Polysaccharides of the Plant Cell Wall

A mature plant cell contains a primary and a secondary cell wall. The polysaccharides of the primary cell wall are cellulose, hemicelluloses and pectin, whereas the secondary cell wall contains cellulose, hemicelluloses, a little pectin and lignin ([3], and ref. therein). The secondary cell wall is produced when the cell stops expanding and is located between the primary cell wall and the plasma membrane.

Dicots and non-commelinoid monocots contain type I primary cell walls, while members of the Poaceae family have type II primary cell walls. The hemicelluloses of type I cell walls consist mainly of xyloglucans, whereas type II walls contain mixed-linkage glucans and glucuronoarabinoxylans [4]. The breakdown of type I and II cell walls generate different elicitor-active oligosaccharides (Table 1). For the structural organization of the different types of cell walls, we refer to [4,5].

### 2.1. Cellulose

Cellulose is a linear glucose polymer, and most of the polymer is organized in paracrystalline microfibrils with a hydrolysis-resistant structure which facilitates its high mechanical strength. Unlike glycogen or starch, the glucose moieties in cellulose are linked via β-1-4 glycosidic bounds. Since every other glucose moiety is inverted, the hydroxyl groups of C1 and C4 are in the same plane. Parallel arrangement of the cellulose molecules allows the formation of strong hydrogen bonds as well as van der Waals forces between the chains, and 18 cellulose molecules have been proposed to assemble to a microfibril. Their hydrophobic surfaces interact with xyloglucans, whereas the interaction with pectin occurs mainly via the hydrophilic surface areas (cf. [23,24] and ref. therein).

Cellulose synthase (CESA) synthesizes cellulose, and cellulose synthase-like (CSL) enzymes synthesize most hemicellulosic polysaccharides in plants [25]. Synthesis of the cellulose polymer occurs at large transmembrane CESA complexes (CSCs) which consist of CESA hexamers and can be visualized as sixfold symmetrical rosettes at the plasma membrane. CESAs catalyze the transfer of D-glucose from UDP-D-glucose to the C4-hydroxyl end of the elongating cellulose polymer [26,27,28]. The CSC complex is associated with proteins for regulating cellulose biosynthesis, assembling it at the endomembrane system and translocating it to the plasma membrane where it becomes active (summarized in [5]). The elongating cellulose chains are translocated through the plasma membrane into the apoplastic space (cf. [23,29] for more details), where they assemble to microfibrils. Assembly of the CSCs starts in the Golgi apparatus. The complexes are then translocated to the plasma membrane and positioned at the places where cellulose synthesis is required. This involves a large number of additional regulatory proteins ([23,30] and ref. therein).

In Arabidopsis, CESAs are encoded by a gene family with 10 members, and CESA1-3, -5, -6 and -9 are involved in primary wall synthesis, while CESA4, -7 and -8 are required for secondary cell wall biosynthesis [31,32]. Besides knock-outs, mutations in phosphorylation sites, e.g., of CESA1, also influence cell expansion and bidirectional mobility of the CSC subunits [33]. An important regulatory protein is CELLULOSE SYNTHASE INTERACTIVE1 (CSI1) which mediates CSC association with cortical microtubules [34]. The CC1/2 N-terminal domain in CSI is responsible for the connection of the CSCs to the cortical microtubules [35,36], and *csi1* mutants are impaired in the dissociation of the CSC from the microtubules to the plasma membrane [37,38]. The CSI1 function for CSC formation is regulated by multiple phosphorylation events [30], as well as its trafficking to and positioning in the plasma membrane. CSI1 is also involved in the mobility of the complex in the plasma membrane and its recycling via clathrin-mediated endocytosis [38]. Numerous other regulatory proteins participate in the assembly of CSC at the endoplasmatic reticulum (ER), its translocation to the plasma membrane via exocytosis, the positioning in the membrane and control of its activity, as well as its recycling via endocytosis [39].

In general, non-lethal impairments in cellulose biosynthesis due either to mutation or to chemical treatments (e.g., isoxaben) activate compensatory repair mechanisms which range from the stimulation of cellulose biosynthesis to synthesis of other cell wall oligomers including pectin and lignin, the activation of defense genes, disease resistance responses, reactive oxygen species (ROS) production and synthesis of secondary metabolites or phytohormones, preferentially jasmonic, salicylic and abscisic acids (JA, SA and ABA, respectively), or ethylene (summarized in [5]). For instance, an Arabidopsis mutant impaired in CESA3 is more resistant to pathogens, perhaps due to its activated immune system. The *ixr1/cev1* (*isoxaben resistant1/constitutive expression of VEGETATIVE STORAGE PROTEIN1*) mutant which is resistant to the chemical cellulose synthase inhibitor isoxaben shows constitutive ethylene and JA signaling, which results in better resistance to pathogens [40,41,42]. Arabidopsis mutants which are impaired in the biosynthesis of CESA4, -7 and -8 for cellulose biosynthesis of the secondary cell wall show enhanced resistance to several necrotrophic fungi and the bacterium *Ralstonia solanacearum* [41,42,43,44]. Interestingly, the *irx1-6* mutant also shows enhanced resistance to abiotic stresses (salinity and drought), presumably because the ABA signaling pathway was activated [42,44,45]. Likewise, the ABA mutant *aba1-6* accumulates less cellulose in its cell wall [41,46], which confirms a link between cell wall integrity (CWI) and ABA signaling. Douchkov et al. [47] observed local cell wall reinforcement upon attempts of pathogens to penetrate the barley host with a silenced CSL-D2. In conclusion, the mutant analyses demonstrate that CWI, immune and abiotic stress responses are connected. Often, the activated immune system has severe consequences for plant growth due to the manipulation of the growth–defense trade-off [40,41,48,49,50,51,52,53].

### 2.2. Hemicelluloses 

Hemicelluloses are composed of diverse sugars, mainly the five-carbon sugars xylose and arabinose and the six-carbon sugars glucose, mannose and galactose. The most abundant hemicellulose in type 1 primary cells are xyloglucans, which consists of a β-1-4-glucan backbone with α-1-6 xylosylation (cf. [54] for detailed description of xyloglucan biosynthesis). The xylans, xyloglucans and (gluco)mannan backbone of β-1,4-linked sugars form an equatorial linkage configuration [55]. The xylose residues of xyloglucans are branched with different patterns of arabinose, fructose, galactose and acetylation that vary between species [54,56]. In contrast to the linear cellulose, hemicelluloses have an amorphous structure, and up to 35% of the xylose residues in angiosperm hemicelluloses can be acetylated. Hemicelluloses constitute roughly one-third of the cell wall biomass, and—comparable to cellulose biosynthesis—all polymerization reactions start with sugar nucleotides at the Golgi apparatus. The detailed steps and known enzymes involved in the polymerization reactions are summarized in [54,56,57]. Compared to cellulose biosynthesis, the biosynthesis involves more enzymes due to the incorporation of different monomeric sugars and the generation of various linkages. The mannan chain backbone is synthesized by mannan synthases, which are A and D members of CSL. The xyloglucan backbone is synthesized by glucan synthases, and they are C members of CSL. A xylan synthase facilitates the addition of xylose to the backbone. The galactose side-chains of some mannans are added by galactomannan galactosyltransferases. Mannans can be acetylated by a not yet identified mannan O-acetyltransferase. The hemicelluloses are transported from the Golgi apparatus to the plasma membrane where the polysaccharides are secreted into the apoplast via Golgi vesicles. For details about the different types of plant hemicelluloses, hemicellulose-specific nucleotide sugar substrates required for their synthesis, key transporters and the biosynthesis pathways, we refer to a recent review by [58].

Hemicellulose polysaccharides influence shape, assembly, architecture and properties of the primary cell wall and interact with cellulose to affect the deposition and bundling of cellulose fibrils [1,59,60]. The xyloglucan endotransglucosylases/hydrolases, which catalyze the polymerization and cleavage of xyloglucan molecules, are key players in cell wall remodeling, elasticity, ductility and reconstruction [61]. Recently, Yu et al. [59] identified a patterned β-galactoglucomannan which shows remarkable similarities to xyloglucan. 

Hemicelluloses of Poaceae and genus *Equisetum* contain mixed-linkage glucans (Table 1). Biochemical and genetic studies showed that the biosynthesis and degradation of mixed-linkage glucans share similarities with those of cellulose, as discussed in Kim and Brandizzi [62]. CSL-F/H family members play a crucial role in biosynthesis, and it has been suggested that, in particular, the F family members have experienced a recent evolutionary hub in Poaceae [63]. Degradation to elicitor-active mixed-linkage glucan oligomers occurs by lichenases (cf. [62]).

Besides mixed-linkage glucans, glucuronoarabinoxylans with linear backbones of β-1,4-xylose residues that may be substituted with α-1,2-linked (4-O-methyl)-glucuronic acid, α-1,3-linked arabinofuranose and sometimes acetylation at the O-2 and/or O-3 positions are abundant non-cellulosic polysaccharides (xylans) in grass cell walls [64]. Little is known about their biosynthesis, function and degradation. Kozlova et al. [65] proposed a model for maize roots in which the mixed-linkage glucans serve as a gel-like filler of the space between the separating domain of the glucuronoarabinoxylan and the cellulose microfibrils. The breakdown of arabinoglucans generates elicitor-active arabinoxylan-oligosaccharides [17] (Table 1).

Mutants directly or indirectly affected in hemicellulose and xylose biosynthesis show severe alterations in their immune responses and susceptibility to pathogens (overview in [41,66,67,68,69,70,71,72]). Hemicelluloses can be acetylated, and mutation in polysaccharide O-acetylation results in altered resistance responses in various pathosystems. One of the examples is ESKIMO1 (ESK1), a polysaccharide O-acetyltransferase involved in xylan acetylation [44,73]. The impairment in xylan acetylation was accompanied by tolerance of the mutants against abiotic and biotic stress [41,44,73,74,75]. This demonstrates that not only impaired polysaccharide biosynthesis but also post-translational modifications activate repair and (a)biotic stress tolerance responses.

### 2.3. Pectin

The main component of pectin is galacturonic acid (Table 1). A linear chain of galacturonic acid monomer units with an α-(1-4)-glycosidic bond builds the backbone which can be interrupted by α-(1-2)-rhamnopyranose units to form side chains consisting of galactose, glucose, mannose and xylose units. The pectin matrix is composed of primarily homogalacturonan (HG), the substituted rhamnogalacturonan-I (RG-I) and rhamnogalacturonan-II (RG-II) [2,76]. HG is a linear polymer of α-(1–4)-linked galacturonic acid, which is modified by methylesterification of the C6 carboxyl group and acetylation on C2 and/or C3 of galacturonic acid residues [77]. The linear chains of HGs branch off with xylose or apiose from the backbone. RG-I pectins contain a backbone of 1.4-linked galacturonic acid and 1,2-linked rhamnose, from which sidechains of galactose and arabinose branch off. RG-II shows a higher degree of branching and contains only galacturonic acid units in its backbone. Synthesis of the complex pectin structure requires at least 67 different glycosyltransferases, methyltransferases and acetyltransfereases [78]. Pectin is synthesized in the Golgi cisternae in a highly methylesterified and acetylated form [2].

### 2.4. Proteins Associated with the Plant Cell Wall

About 10% of the cell wall biomass derives from proteins, which are required, e.g., for cell wall modification, recognition of cell-external stimuli and initiation of responses to biotic and abiotic stresses. Recently, San Clemente et al. [79] compared the proteomic pattern of cell wall extracts from various plant species and proposed a core cell wall proteome, which comprised: (i) glycoside hydrolases and pectin methyl esterases, (ii) class III peroxidases, (iii) aspartate, serine and cysteine proteases, (iv) non-specific lipid transfer proteins, (v) fasciclin arabinogalactan proteins, (vi) purple acid phosphatases and (vii) thaumatins. The specific role of some proteins during CWI signaling will be described below.

## 3. Polysaccharide Degradation

Beneficial and pathogenic microorganisms, insects and nematodes contain an arsenal of cell wall-degrading enzymes (CWDEs), including those which degrade crystalline regions in polysaccharides (cf. below). The microbial enzymes are involved in establishing beneficial symbiotic interactions, virulence, or generation of short-chain sugar molecules for microbial digestion [80,81,82,83,84]. They have their own evolutionary history and adaptation mechanism to their hosts. Plants contain proteins which inhibit the microbial CWDEs, but also microbes can release proteins and chemical compounds which interfere with plant CWDEs (cf. below). Breakdown products of the plant cell wall polysaccharides can also be generated by abiotic stresses, mechanical injury, or plant internal signals, such as hormones controlling growth, cell expansion or re-arrangement or cell division. Classical model systems for cell wall rearrangements controlled by internal signals are root hair growth or pollen tube development, female tissue recognition, germination, polar growth or phototropism (e.g., [85]). Breakdown products of the wall polysaccharides can function as DAMPs in the apoplast and are recognized by pattern recognition receptors (PRRs). Genetic techniques which change the cell-wall-modifying enzyme pattern allow the manipulation of the cell wall structure and the generation of new elicitor-active compounds or compound combinations [86].

The amorphous regions of cellulose are better accessible to hydrolytic enzymes than the paracrystalline regions. They are degraded by four types of enzymes. β-1,4-endoglucanases hydrolyze the internal bonds of the cellulose chain. Exoglucanases are mainly cellobiohydrolases which generate cellobiose units. They are cleaved to glucose monomers by β-glucosidases. Metazoans lack cellulases and thus cannot degrade cellulose, whereas plants, fungi and bacteria and a few insects and nematodes contain enzymes for cellulose degradation [87]. For instance, cellulases from *Magnaporthe grisea* and *G. rostochiensi* contribute to host penetration and tissue invasion [88,89], and bacterial cellulases, e.g., from *Clavibacter michiganensis*, participate in virulence in tomato [90]. The microbial cellulases can operate in concert with lipid polysaccharide monooxygenases (LPMOs, cf. below) (cf. [87,91]). Fungal cellulases contain a carbohydrate-binding module, and microbial cellulases are often expressed in response to plant infections. In all instances, degradation of cellulose results in the generation of elicitor-active cello-oligomers of different lengths [6] (Table 1), while the end product glucose may interfere with the primary metabolism and serves as an energy source for the microbes.

When compared to cellulose, hemicelluloses are degraded faster, and the breakdown products are a mixture of quite diverse oligosaccharides. Hemicellulose-degrading enzymes are usually named according to their substrates and are also endo- or exoenzymes (L-arabinanases, D-galactanases, D-mannanases and D-xylanases). Xylan, an abundant hemicellulose, is cleaved by a battery of xylanolytic enzymes, the most important ones are the endo-β-1,4-xylanases [92]. Xyloglucan breakdown products activate signaling cascades which are similar to those activated by oligogalacturonides (OGs), the breakdown products of pectin (Table 1) [21]. Degradation of mixed-linkage glucan polysaccharides from, e.g., Poaceae hemicellulose, generates the elicitor-active mixed-linkage β-1,3/β-1,4 glucan oligosaccharides ([7], and ref. therein) (Table 1).

Pectin is degraded by hundreds of pectic degrading enzymes of either plant or microbial origin, which are often activated in response to defined threats. The carboxyl group of galacturonic acid is often converted into a methyl ester, and the side group needs to be removed by methylesterases before pectate lyases or polygalacturonases can degrade the polymer. In addition, pectin acetylation occurs during exocytosis and incorporation of pectin into the cell wall, and apoplastic de-acetylation leads to increased resistance to pathogens [93,94,95,96] due to faster pectin degradation. This results in the accumulation of OGs, oligomers of α-galacturonosyl residues linked by α-1,4-glycosidic linkages, which are perceived by the PRR WALL-ASSOCIATED KINASE1 (WAK1) (Table 1). The stimulated immune system restricts pathogen growth (e.g., [93,94]).

Furthermore, the pectin methylesterification status is strongly altered in response to infection and is controlled by pectin methylesterases (PMEs). In addition, their activity is post-transcriptionally regulated by pectin methylesterase inhibitors (PMEIs) [97]. In Arabidopsis, the endogenous PMEI10, PMEI11, PMEI12 and PMEI17 are induced during *B. cinerea* infection. *PMEI* expression is strictly regulated by JA and ethylene signaling, while *PMEI11* expression is controlled by PME-related DAMPs, such as OGs [97,98].

Plants also respond to microbial polygalacturonases (PGs) by synthesizing polygalacturonase-inhibiting proteins (PGIPs; [99]) which are specific for microbial or insect PGs. Consistent with their inhibitory roles, PGIP over-expressor lines are more resistant to necrotrophic fungi and bacteria [100,101]. Furthermore, the degree of methylesterification of pectin reduces accessibility of the degrading enzymes to pectin which also influences fungal penetration [102].

### 3.1. Degradation of Polysaccharides by Lytic Polysaccharide Monooxygenases (LPMOs)

Valente et al. [103] discovered that the expression of a *GLYCOSYL HYDROXYLASE61* gene encoding an LPMO from the hemibiotrophic ascomycete *Pyrenochaeta lycopersici* of tomato correlated to the development of the induced disease symptoms. Since this report in 2011, the number of studies on LPMO enzymes has increased exponentially. LPMOs are found in different Ascomycota and Basidiomycota, many pathogenic microbes and are involved in cell wall degradation and defense gene activation in their hosts [104,105]. LPMOs oxidatively cleave the glycosidic bonds in carbohydrate polymers, preferentially cellulose and chitin, even in their crystalline regions [91,104,105,106,107,108,109]. *LPMO* genes of major fungal pathogens are upregulated during pathogen invasion and transmission from a biotrophic to a necrotrophic interaction, and this correlated with disease development. Among the different LPMO families, the AA9 family is exclusively found in fungi, and they cleave mainly cellulose (for more information, cf. [104,110,111]). Zarrattini et al. [8] showed that LPMO-induced cellulose degradation results in the generation of DAMPs which trigger innate immune responses leading to increased resistance to the necrotrophic fungus. Since the LPMOs are able to oxidatively cleave cellulose, they generate a unique mixture of oxidized and native oligosaccharides [8]. The LPMO-mediated stimuli induce the expression of two receptors, STRESS-INDUCED FACTOR2 and -4 (SIF2 and SIF4), and they might interact with BRI1 ASSOCIATED RECEPTOR KINASE1 (BAK1) and THESEUS1 (THE1) ([8], cf. below). LPMO enzymes of other families are also found in arthropods and oomycetes. Besides the production of novel DAMPs, LPMOs have a huge potential for decomposing waste cellulose and chitin [112,113,114].

### 3.2. Berberine Bridge-Enzyme-like (BBE-like) Proteins Oxidize OGs and Cello-Oligomers to Prevent Hyper-Immunity

Overaccumulation of DAMPs may cause hyper-immunity and has negative effects on the growth–defense trade-off. To maintain homeostasis and restrict immune responses, several DAMPs, such as OGs and cello-oligomers, can be oxidized by BBE-like proteins. Oxidation inactivates the DAMP activity, but also makes them a less desirable food source for microbial pathogens [115]. Benedetti et al. [116] showed that four Arabidopsis BBE-like proteins (called OG OXIDASE (OGOX)1–4) oxidize OGs and impair their elicitor activity. OGOX1 (At4g20830) is a sulphite-sensitive H_2_O_2_-producing enzyme that displays maximal activity on OGs with a degree of polymerization >4. Oxidized OGs display not only a reduced capability of activating immune responses but are also less hydrolysable by fungal PGs. Plants overexpressing OGOX1 are more resistant to *Botrytis cinerea* [115]. Locci et al. [11] showed that the Arabidopsis BBE-like protein At4g20860, which is expressed coordinately with OGOX1 during immunity, specifically oxidizes cello-oligomers with a preference for cellotriose and longer fragments (n4–n6). The preference for cellotriose points to a specific role of this cellulose breakdown product in the immune response and is in line with the observation that cellotriose is the most effective cello-oligomer in inducing immune signaling events, such as cytoplasmic Ca^2+^ elevation and production of ROS [10]. Since oxidized cello-oligomers show negligible elicitor activity and are less easily utilized as a carbon source by *B. cinerea*, plants overexpressing this cello-oligomer oxidase (At4g20860) displayed enhanced resistance to the pathogenic fungus. Thus, the capacity to oxidize and impair the biological activity of cell-wall-derived oligosaccharides seems to be a general trait of the family of BBE-like proteins, which may serve to homeostatically control the level of DAMPs to prevent their hyperaccumulation [11]. The involvement of the remaining members of the BBE-like protein family with 27 members in Arabidopsis need to be studied in more detail [115]. Messenlehner et al. [117] investigated the catalytic features of AtBBE-like 15 in Arabidopsis and identified conserved amino acids required for alcohol oxidation. This might help to identify substrates for the not yet studied BBE-like protein members. However, there are also species-specific differences among BBE-like proteins. For instance: multiple sequence analysis showed that the active site composition of AtBBE-like 28 is only present in the Brassicaceae [118]. Furthermore, BBE proteins are present in plants, fungi and bacteria [119], and the function of the microbial enzymes in beneficial and pathogenic plant/microbe interactions has been little studied. Liu et al. [120] showed that the *Magnaporthe oryzae* BBE-like protein MoSef1 is an apoplastic virulent factor that inhibits the host immune response. In conclusion, it is reasonable to assume that more plant and microbial BBE-like proteins participate in the growth–defense trade-off.

## 4. Perception of Cell Wall Breakdown Products

Breakdown products of the plant cell wall polysaccharides function as DAMPs and are recognized by PRRs. They can recruit additional apoplastic or membrane-bound proteins to assemble PRR complexes. Activation of the immune system, similar to PAMPs/MAMPs, has a strong influence on the “growth-defense trade-off” [41,121,122]. Furthermore, as components of the cell wall surveillance system, they activate cell wall repair processes by stimulating the biosynthesis of one or more polysaccharides. OGs from pectin [123], short-chain xyloglucans [21], mixed-linkage glucans [7] and arabinoxylan [17] and mannan oligosaccharides from secondary cell walls of gymnosperms [22] were identified and characterized as elicitor-active compounds in various species (Table 1).

More than 600 genes for receptors, receptor kinases (RKs) and receptor-like kinases (RLKs) are present in Arabidopsis [124,125,126]. Besides RKs, receptor proteins without kinase domain or apoplastic receptor proteins with ligand-binding properties and membrane anchors are common, and they recruit interaction partners for the missing epitopes. While much is known about peptide perception, carbohydrate perception and that of carbohydrate-bound ligands are not well characterized in plants, although many proteins with carbohydrate-binding motifs are present in Arabidopsis [125]. Among them are lectins, including the Lysin Motif (LysM) proteins [127], cysteine-rich and wall-associated (receptor) kinases, [128], Crinkly-Like (CR4L) proteins and malectin (MAL) domain-containing RLKs [126,129]. Besides breakdown products of its own cell wall, those from microbial cell walls are also recognized by plant cells. The well-characterized β-1,4-acetylglucosamine from the fungal cell wall is bound by the CHITIN ELICITOR RECEPTOR KINASE1 (CERK1) in Arabidopsis [19,130,131]. Mixed linkage β-1,3/1,4-glucan oligosaccharides are generated during the breakdown of 1,3/1,4-glucans of the cell wall polysaccharides of fungi, oomycetes and some bacteria [13,14,132,133], but also of monocot (Poaceae) cell walls [20]. They induce immune responses in different plant systems, including rice, barley, Arabidopsis, tomato and pepper [18,20,134]. Peptidoglycan from the bacterial cell wall [135], mannan oligosaccharides [58] and fructan [136] also elicit immune responses in plants. However, their detection systems have not yet been identified (cf. [134]).

### 4.1. OG Perception by WAKs

**Figure 1:**
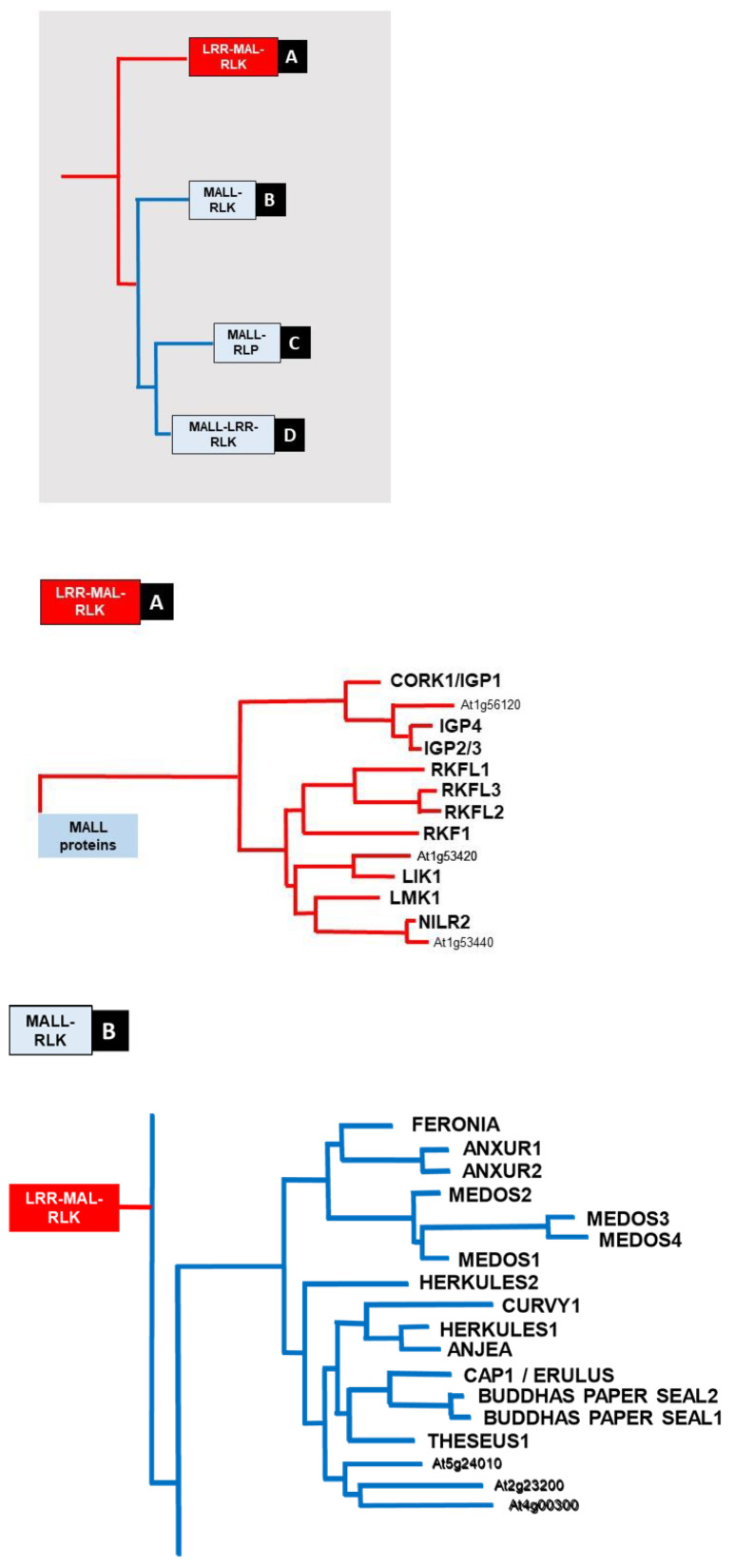

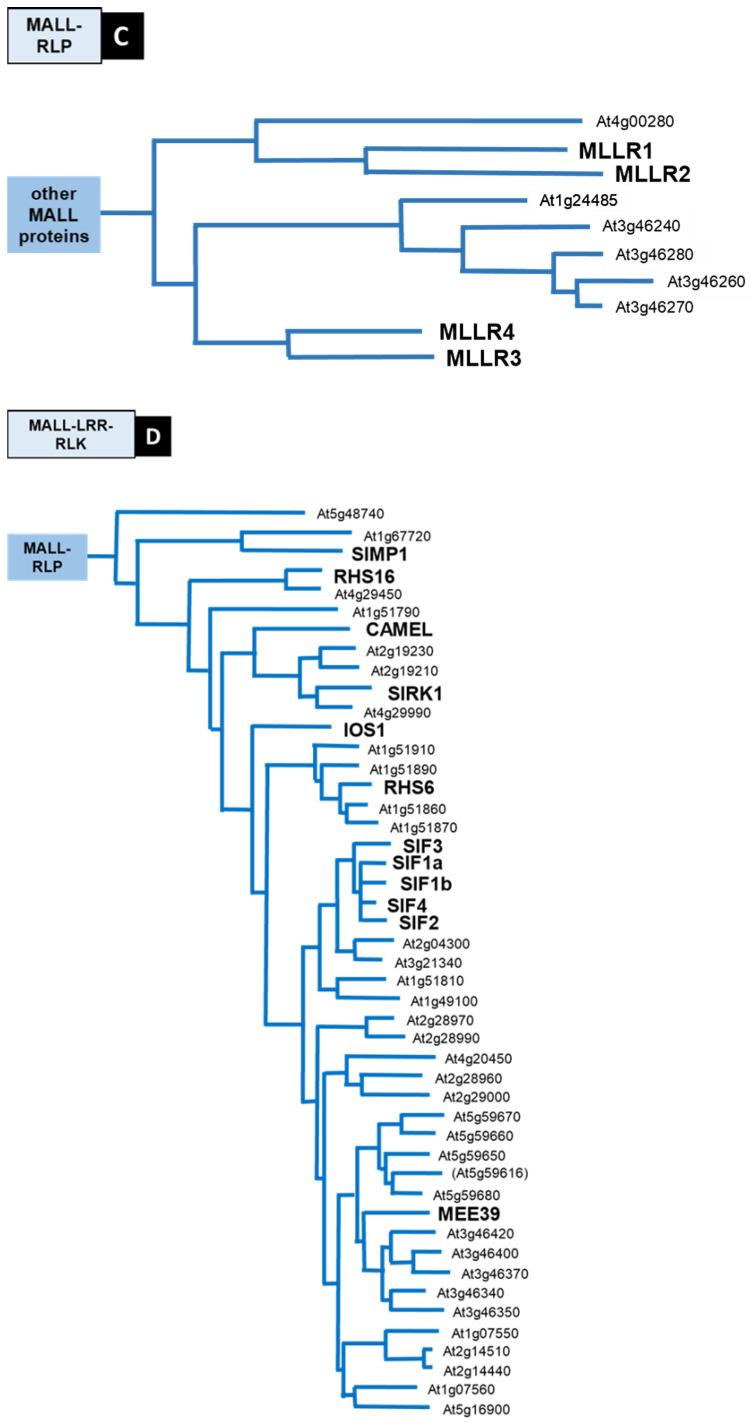
Phylogenetic tree of MAL- and MALL-proteins. The tree shows one branch with MAL domain (PF11721)-containing proteins (red, **A**) and three branches with the distantly related MALL domain (PF12819)-containing proteins (blue, **B**–**D**). For details, cf. Yang et al. [134]: (**A**) LRR-MAL-RLKs; (**B**) MALL-RLKs; (**C**): MALL-RLPs; (**D**) MALL-LRR-RLKs. Protein (and abbreviations) described in the literature are in bold (and discussed in the text). For the others, the Arabidopsis protein numbers are given. For details, cf. TAIR-homepage (arabidopsis.org).

WALL-ASSOCIATED KINASE (WAK) receptors are PRRs for the perception of the pectin-breakdown products OGs [137], but also the FERONIA (FER, At3g51550) RLK, which belongs to a different protein family (Figure 1), has been shown to bind highly demethylesterified pectin [138]. WAKs are well-characterized in many plant species and interact with OGs of 10–15 monomers [115,122,139,140,141,142,143,144,145]. Recombinant WAK1 and WAK2 bind to the de-esterified, charged galacturonic acid backbone of pectins in vitro and induce immune responses [146]. However, WAK1 has also been implicated to modulate the sugar metabolism [147]. Binding of the linear pectin backbone to WAKs requires Ca^2+^ and ionic conditions for the formation of Ca^2+^ bridges. Chemical modification of the reducing end of the OGs diminished their eliciting activity but did not hinder either dimerization or binding to WAK1 [148]. Brutus et al. [137] performed domain swap experiments and confirmed OG perception by the WAK ectodomain and signaling through the cell-internal kinase domain.

The WAK/WAK-like (WAKL) gene family in Arabidopsis consists of 26 members [149,150]. *WAK* family members are preferentially expressed in green tissues, whereas *WAKL* genes are mainly expressed in roots and flowers. WAK22 is involved in resistance against *Fusarium oxysporum* [151] in Arabidopsis, and the involvement of WAKLs in disease resistance has also been shown for other plant species [152,153,154,155,156,157]. In tomato, activated WAK1 is important for later but not early pattern-triggered immunity (PTI) responses and forms a complex with FLAGELIN-SENSING2/3 (FLS2/FLS3) [158]. *WAK1* knock-out plants of tomato developed fewer callose deposits than wild-type plants but retained early PTI responses [158]. Expression of *WAK1*-targeting RNAi constructs in rice resulted in dwarfism [159]. These examples show the participation of WAKs in PTI responses and interference with the growth/defense balance. Hou et al. [160] suggested versatile roles for *WAKL4* in mineral nutrition responses, and *WAKL5* and *WAKL7* are induced by wounding [150]. The precise function of the individual members is difficult to predict because of redundancy among the individual family members. Tripathi et al. [161] identified 91 *WAK/WAKL* genes in barley, and more than 50 *WAK/WAKL* genes are present in the *Gossypium arboreum*, *G. raimondii* and *G. hirsutum* genomes [162].

The apoplastic GLYCINE-RICH PROTEIN3 (GRP3) binds to the extracellular domain of Arabidopsis WAK1 [163]. The WAK1/GRP3 complex associates with the KINASE-ASSOCIATED PROTEIN PHOSPHATASE (KAPP) [164,164], and the trimeric complex negatively affects defense responses induced by OGs [164]. GRP3 and WAK1 are also involved in cell elongation, root development and aluminum resistance (reviewed in [165,166,167,168]). Apparently, the activated WAKs induce a quite diverse set of cellular responses, which also depend on WAK-interaction partners.

### 4.2. Perception of Cellulose Breakdown Products and Mixed Linkage Glucan-Derived Oligosaccharides

Cellulose degradation products (cello-oligomers) induce a variety of defense responses in grapevine (*Vitis vinifera*) cells, as first described by Aziz et al. [12]. Locci et al. [11] showed that cellotriose and, to a lesser extent, cellotetraose to cellohexose induce ROS production, phosphorylation of mitogen-activated protein kinases (MAPKs) and other proteins, as well as the activation of defense gene expression. Souza et al. [9] demonstrated that cellobiose triggers a signaling cascade that shares similarities with responses to well-known elicitors, such as chitooligomers and OGs. In contrast to other known P/DAMPs, cellobiose stimulates neither ROS production nor callose deposition. In addition, transcriptome profiles were very similar after cellobiose and OG treatments [9,123]. Perception of cellulose-derived oligomers may participate in CWI surveillance and represents an additional layer of signaling following plant cell wall breakdown during cell wall remodeling or pathogen attack [9]. Co-infection experiments of cellobiose with other PAMPs and DAMPs showed that these elicitors probably co-occur at infection sites, leading to synergistic increases in gene expression. Johnson et al. [10] found that cellotriose is the elicitor-active cell wall moiety released by the endophytic beneficial fungus *Piriformospora indica* into its growth medium. It induces rapid cytoplasmic Ca^2+^ elevation in Arabidopsis and tobacco root cells. Cellotriose also activated a mild defense-like response, including the production of ROS, changes in membrane potential, and the expression of genes involved in growth regulation and root development. It acted synergistically with chitin. Induction of the Ca^2+^ response by cellotriose and activation of the downstream responses requires the poly(A) ribonuclease (AtPARN; At1g55870) which degrades the poly(A) tails of specific mRNAs in roots. How PARN is integrated into cello-oligomer signaling is not clear. Thus, evidence for cello-oligomer-induced signaling in plants has been reported for several systems [87].

More recently, Tseng et al. [6] and Martín-Dacal et al. [7] identified leucine-rich repeat (LRR)-MAL domain containing RLKs required for cello-oligomer signaling in Arabidopsis by isolating mutants which do not show cytoplasmic Ca^2+^ elevation in response to the application of cello-oligomers [6] or mixed-linkage glucans [7].

Tseng et al. [6] showed that the CELLOOLIGOMER-RECEPTOR KINASE1 (CORK1) (At1g56145), a MAL containing RLK, is preferentially activated by cellotriose. CORK1 is required for cello-oligomer-induced cytoplasmic Ca^2+^ elevation, ROS production, MAPK activation, cellulose synthase phosphorylation and the regulation of CWI-related genes, including those involved in biosynthesis of cell wall material, secondary metabolites and tryptophan. Phosphoproteome analyses identified early targets involved in signaling, cellulose synthesis, the ER/Golgi secretory pathway, cell wall repair and immune responses. Two conserved phenylalanine residues in the MAL domain are crucial for CORK1 function. Mainly based on the analyses of downstream phosphorylation events, the authors concluded that CORK1 is required for CWI and immune responses activated by cellulose breakdown products. Using isothermal titration calorimetry assays, Martín-Dacal et al. [7] provided direct evidence for binding of cellotriose/cellopentaose to the ectodomain of CORK1, which they named IGP1 (IMPAIRED IN GLUCAN PERCEPTION1).

Besides cello-oligomers, Rebaque et al. [18] showed that oligosaccharides derived from mixed-linkage β-1,3/1,4-glucans, which are present in plant and microbial cell walls, are perceived by *A. thaliana*. They identified a trisaccharide, β-d-cellobiosyl-(1,3)-β-d-glucose, as the smallest structure triggering strong PRR-triggered immune responses. Two LRR-MAL domain-containing RLKs (IGP2/3 and IGP4; At1g56130 and At1g56140), which are closely related to the cellotriose-binding CORK1/IGP1 receptor (Figure 1A) are required for the induction of cytoplasmic Ca^2+^ elevation by the mixed-linkage glucan derived oligosaccharides [7]. While cellotriose and cellopentaose bind to the ectodomain of CORK1/IGP1, the mixed-linkage glucan did not bind. The PTI responses elicited by the mixed linkage glucan oligosaccharides, but not by the cello-oligomers, are partially dependent on LysM PRRs CERK1, LYK4 and LYK5, as they were weaker in *cerk1* and *lyk4 lyk5* mutants than in wild-type plants. There appears to be a so far not well understood specificity for perception of the cello-oligomers and mixed-linkage glucans, and Martín-Dacal et al. [7] proposed that that CORK1/IGP1 is the receptor for cello-oligomers from cellulose and At1g56140 might function as a co-receptor in the sensing complex for cellulose and mixed-linkage glucan-derived oligosaccharides. Furthermore, besides *cork1/ipg1*, the mutants impaired in *at1g56130* and *at1g56140* were also impaired in cellotriose perception. Models of the structure of the three LRR-MAL-RKs showed that the CORK1/IGP1 kinase domain is different from that of the two others (IGP2/3 and -4) due to an extra loop seen in the extracellular part of its complete structure, whereas the catalytic sites are nearly indistinguishable in the three proteins [7]. These results add a novel and so far not investigated LRR-MAL-domain containing RLKs from Arabidopsis to the group of PRRs which perceive oligosaccharide fragments. As discussed by both groups [6,7], crosstalk of the novel perception systems with others requires further investigations.

Interestingly, orthologs of the LRR-MAL-RKs identified in Arabidopsis exist in monocots (rice and sorghum), and several studies showed that mixed-linkage glucans induce cellular responses in cereals [18,20]. Whether cello-oligomer perception is important for agriculturally interesting plants is unclear.

## 5. MAL Domain RLKs

In bacteria, insects and animals, carbohydrtate-binding proteins with a MAL domain recognize short-chain sugar moieties, although not cello-oligomers. Schallus et al. [169] found a novel carbohydrate-binding protein of the ER in *Xenopus laevis* involved in early steps of protein N-glycosylation. The *Xenopus* protein has a homolog in human, and it reveals a close structural similarity to carbohydrate binding modules of bacterial glycosylhydrolases. It binds to maltose and related oligosaccharides and was named “malectin”. Malectins are about 190 amino-acid long proteins and part of the oligosaccharyltransferase complex in the ER, which bind to N-linked glucans of intermediate proteins in the secretory pathway, recognize misfolded glyopropteins and target them for degradation. Therefore, malectins participate in protein quality control of ER proteins and those sorted via the ER secretory pathway [170,171,172,173,174]. In vitro studies demonstrate that besides the disaccharide maltose with two α-1,4-bound glucose moieties, malectins also bind nigerose with two α-1,3-bound glucose moieties [7].

In rice, the MALECTIN LIKE-DOMAIN PROTEIN *MLD1* located in the ER may play a similar role to its mammalian homologs in glycoprotein quality control, thereby regulating cell death and immunity [175]. However, most MAL-domain-containing proteins in plants are RLKs in the plasma membrane with their MAL regions in the extracellular space. Considering the ER secretory pathway, the apoplast of a cell is topologically similar to the ER lumen, and membrane protein domains facing the ER lumen are exposed to an extracellular environment after the ER-plasma membrane vesicle flow [176]. These properties and the functions of the first two studied MAL-like (MALL)-domain RLKs, THE1 (At5g54380) and FER (At3g51550) [177,178] suggest that the plant MAL/MALL sequences might interact with cell wall polysaccharide breakdown products [126,179,180,181,182,183]. In fact, THE1 and FER, as well as several other family members, such as CURVY1 (CVY1, At2g39360), HERKULES1/2 (HERK1/2 At3g46290 and At1g30570, respectively), ANXUR1/2 (ANX1/2, At3g04690 and At5g28680, respectively), ERULUS (ERU, At5g31350), CORK1/IGP1 and the two unnamed At1g56130 and At1g56140, have been shown or are discussed as regulators of cell expansion, CWI sensing and cell wall repair [6,7,184]. 

The structural organization and evolution of membrane-bound MAL domain-containing proteins distinguish between those with a MAL domain (PF11721) or with a MALL domain (PF12819) structure (http://pfam.xfam.org, accessed on 19 November 2021) [185]. MALL proteins are longer and more variable compared to those with a MAL domain, and they often contain two MALL domains which are located at the N-terminal end of the proteins. In contrast, the MAL domain in the MAL-RLKs is located in the apoplast between an LRR domain at the N-terminus and the transmembrane span. Phylogenetic analyses show that MALL-domain-containing proteins can be classified into MALL-LRR-RLKs, MALL-RL proteins and MALL-RLKs, whereas only one group of MAL-domain-containing kinases with an LRR-MAL-RLK arrangement is found in plants (Figure 1).

### 5.1. MALL-RLKs

**Figure 2:**
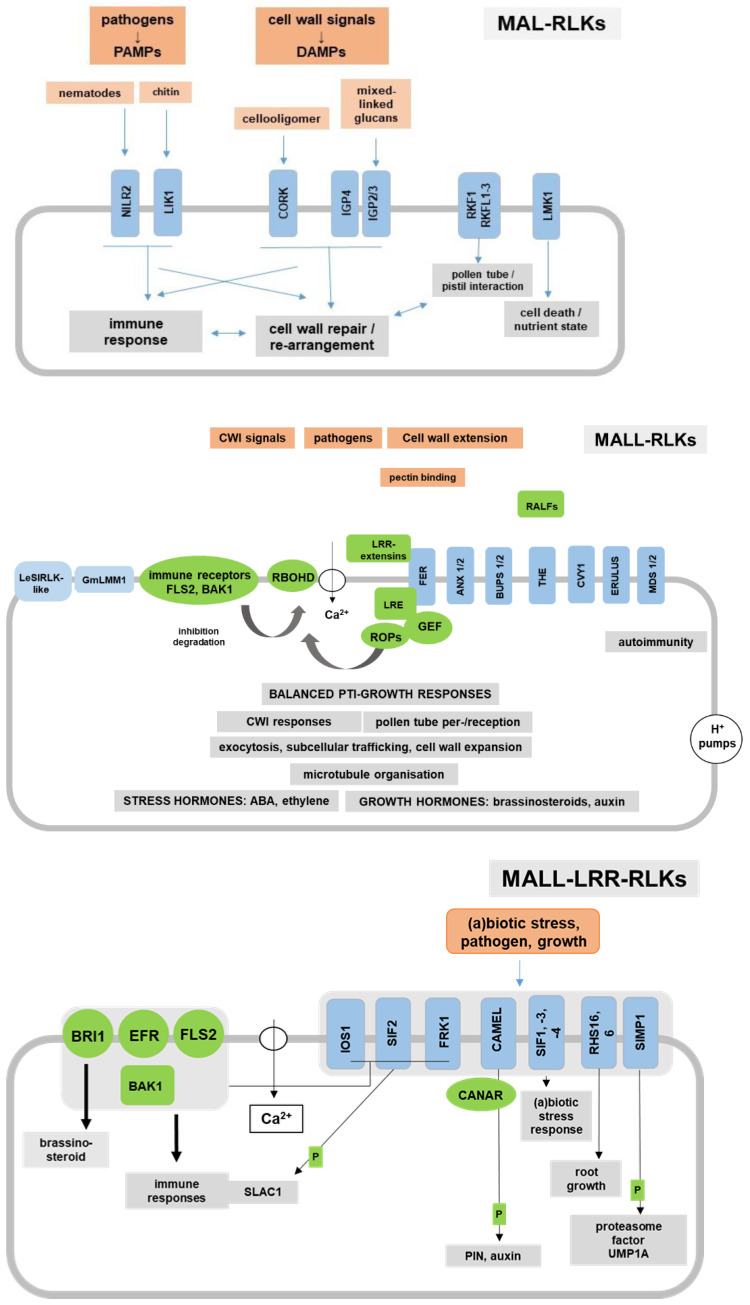
Activation, interaction and signaling of MAL- and MALL-RLKs. They are grouped according to their phylogenetic relationship (Figure 1) into LRR-MAL-RLKs, MALL-RLKs and MALL-LRR-RLKs. Arabidopsis MAL- and MALL-RLKs and GmLMM1 and LeSIRLK-like are in blue. They are activated by apoplastic microorganisms or signals (orange). Interaction partners and downstream target proteins are in green. P means phosphorylation of target proteins. Cellular responses upon activation of the RLKs are in grey boxes. Additional information, abbreviations and references are given in the text.

MALL-RLKs are involved in immune responses, cell wall extensibility and cell expansion, CWI sensing, developmental processes such as pollen tube reception or tip growth and hormonal and abiotic stress responses [134,186,187] (Figure 1 and Figure 2). A total of 8 members of the 18 MALL-RLKs, also named the *Catharanthus roseus* RLKs (CrRLK1L), are well characterized and induce Ca^2+^ dynamics, ROS production and exocytosis of cell wall material and defense compounds: FER, ANX1 and -2, HERK1 and -2, the Ca^2+^-ASSOCIATED PROTEIN KINASE1 (CAP1/ERU), THE1 and CVY1 (Figure 2). Their genes are expressed in different tissues and during various developmental stages (e.g., root hair formation, vegetative growth in adults and seedlings, gametophytes during fertilization) [187]. Nissen et al. [187] proposed a model for a common signaling pathway for these MALL-RLKs that is based on their spatial and temporal control of cell wall extensibility throughout the entire plant. It involves the interaction of the activated RKs with glycosylphosphatidylinositol-anchored proteins (GPI-APs), guanine nucleotide exchange factors (GEFs) and Rho-like GTPases (ROPs). ROPs induce NADPH oxidase-dependent ROS production, which affects cell wall components and Ca^2+^ influx. The level of NADPH oxidase activity may control the balance between cell-wall-loosening hydroxyl radicals and cell-wall-rigidifying H_2_O_2_. Increased intracellular levels of Ca^2+^ stimulate the exocytosis of cell wall material and regulate NADPH oxidase activity. Decreased apoplastic Ca^2+^ may lead to cell wall loosening through reduced Ca^2+^ cross-linking of pectin [187].

THE1, like other members of the MALL-RLK family (FER, ANX1/2, BUDDHA’s PAPER SEAL1/2 (BUPS1/2)), binds rapid alkalinization factor (RALF) peptides [188,189,190,191], and the THE1/RALF34 receptor complex regulates lateral root initiation, suggesting that THE1 integrates CWI and growth [188]. RALFs are ubiquitous small cysteine-rich peptides that mediate apoplastic alkalinization by interaction with MALL-RLKs. They are negative regulators of plant immune responses since their interaction with MALL-RLKs inhibits the formation of the signal receptor complexes. Ortiz-Morea et al. [128] showed that RALF peptides, LORELEI (LRE)-like GPI-APs and cell-wall-associated LRR extensins, coordinate with MALL-RLKs to orchestrate immunity mediated by PRRs and intracellular nucleotide-binding LRR receptors.

Likewise, FER interacts with RALFs and controls root hair development, pollen tube reception in the female gametophyte and activates brassinosteroid and ethylene signaling [23,178,192,193,194,195]. FER also interacts with RALF peptides from colonizing fungi [196]. FER is involved in CWI maintenance under salt stress [197], and the RLK binds to pectin which induces cytoplasmic Ca^2+^ transients. During growth and morphogenesis, plant cells respond to mechanical stresses resulting from spatiotemporal changes in the cell wall that bear high internal turgor pressure. Tang et al. [137] showed that the FER–pectin complex senses and/or transduces these mechanical forces to regulate microtubule organization through activating the ROP6 signaling pathway [137]. FER is also involved in the phototropin 1-mediated blue light phototropic growth in Arabidopsis [198] and auxin signaling [198]. In addition, Nissen et al. [187] linked the MALL-RLKs ANX1/2 to pectin biosynthesis. Consistent with the inhibitory role of RALFs in plant defense, FER also promotes plant growth and development [196]. FER mutants show higher levels of immunity responses [196], and are more resistant to some bacterial and fungal pathogens [196]. Such a scenario has also been described for strawberry [199]. RALF2 in pear controls pollen tube growth through ROS signaling via the MALL-RLK/MAPK18 module [200]. FER also regulates salt tolerance and abscisic acid (ABA) sensitivity in *Malus domestica* [201]. Gao et al. [202] investigated the contact of the pollen tube with the ovule which triggers Ca^2+^ spiking in the synergids that induces pollen tube rupture and sperm release. This process, termed pollen tube reception, entails the action of three synergid-expressed proteins in Arabidopsis: FER, the GPI-AP LRE and NORTIA (NTA), a transmembrane protein of unknown function. Two pollen-tube-derived RALF peptides as ligands for the FER-LRE co-receptor recruit NTA to the plasma membrane. NTA functions as a calmodulin-gated Ca^2+^ channel required for Ca^2+^ spiking in the synergid. The FER-LRE-NTA trio forms a receptor-channel complex in the female cell to recognize male signals and triggers the fertilization process. Collectively, FER appears to be involved in multiple processes activated by alterations in the apoplastic space.

Phylogenetically related to FER are the two MALL-RLKs, ANX1 and ANX2 (Figure 1). They are required for the maintenance of pollen tube growth (cf. [203]), and their MALL domains have been crystalized [204], but no ligand has been identified yet.

MALL-RLKs appear to play a major role in establishing moderate defense responses, as demonstrated by their inhibition through RALF peptides. Additional support comes from a soybean MALL-RLK called GmLMM1 (LESION MIMIC MUTANT1), which shows the closest homology to the Arabidopsis FER and ANX1 [205]. Mutants of GmLMM1 exhibit enhanced resistance to both bacterial and oomycete pathogens, as well as elevated ROS production upon treatment with flg22. Overexpression of the gene in *Nicotiana benthamiana* severely suppresses flg22-triggered ROS production and oomycete pattern XEG1-induced cell death. GmLMM1 interacts with FLS2 and its co-receptor BAK1 to negatively regulate flg22-induced complex formation between them [205]. The authors conclude that the kinase acts as a molecular switch to control an appropriate immune activation in soybean. 

Furthermore, Sussholz et al. [206] identified a MALL-RLK named SIRLK-like in tomato (a close homolog to the Arabidopsis At5g24010) which interacts with the ETHYLENE-INDUCING XYLANASE2 (EIX2) receptor and represses EIX2-induced immune responses. Knockout of SlRLK-like increased EIX2-induced ethylene production and 1-aminocyclopropane-1-carboxylate synthase gene expression. Co-expression of SlRLK-like with LeEIX2 led to a reduction in its abundance, apparently through an ER-associated degradation process. Interestingly, the SlRLK-like MALL domain is essential and sufficient for the interaction with and modulation of LeEIX2. Moreover, SlRLK-like associated with FLS2, resulting in its degradation and concomitantly a reduction of the flg22-induced ROS burst. In addition, SlRLK-like co-expression with other receptor-like proteins (Ve1 and AtRLP23) also led to a reduction in their abundance. This shows that the MALL domain of SlRLK-like acts as a central regulator of LeEIX2 protein levels, and that SlRLK-like leads to a decreased stability of various PRRs, leading to a reduction in their abundance and resulting in attenuation of defense responses [206]. 

Another example for a modulating function of MALL-RLKs provides MEDOS (MDS)1/LETUM2, MDS2/LETUM1 (Figure 1B) and the LORELEI-LIKE-GPI-ANCHORED PROTEIN1 (LLG1) in Arabidopsis. The three proteins regulate the activation of the nucleotide-binding LRR repeat protein SUPPRESSOR OF MAP KINASE KINASE1 MAP KINASE KINASE2 (MEKK2)2 (SUMM2) and the MEKK2 as regulators of cell death and autoimmunity [207]. MDS1/LETUM2 complexes with MEDOS2/LETUM1 and promotes phosphorylation of MDS2/LETUM1. LLG1 interacts with the ectodomains of the MALL-RLKs and facilitates their transport to the plasma membrane. MEKK2 scaffolds MDS2/LETUM1 and SUMM2 and thus counteracts SUMM2 ubiquitination and degradation which promotes autoimmunity [208].

### 5.2. MALL-LRR-RLKs

Much less is known for MALL-LRR-RLKs although this subfamily contains most of the members of the MALL domain-containing proteins in Arabidopsis (Figure 1D and Figure 2). IMPAIRED OOMYCETE SUSCEPTIBILITY1 (IOS1) [209,210] is critical for BAK1-dependent and BAK1-independent PTI. BAK1 interacts with EFR (the Arabidopsis PRR for the PAMP elf18 derived from the elongation factor thermal unstable (EF-Tu)) and FLS2 [191], as well as BRASSINOSTEROID INSENSITIVE1 (BRI1) to induce brassinolide signaling. BAK1 is also a co-receptor of the STRESS-INDUCED FACTOR2 (SIF2) kinase, one of four SIF proteins in the MALL-LRR-RLK family (Figure 1D). Their genes exhibit temporally and spatially distinct expression patterns and SIF2 overexpression enhanced PAMP-induced immunity and pathogen resistance [211]. SIF2 also participates in stomatal immunity through phosphorylation of SLOW ANION CHANNEL-ASSOCIATED 1 (SLAC1) [212]. Since the kinase controls the expression of the Ca^2+^-signaling-related *PHOSPHATE INDUCED1* gene, Ca^2+^ is likely involved in signal transduction [211]. Finally, *FLG22-INDUCED RECEPTOR-LIKE KINASE 1 (FRK1, At2g19190)* is considered as a PTI marker gene and involved in early defense responses. This suggests crosstalk between these MALL-LRR-RLKs and Ca^2+^/BAK1-dependent immune responses (Figure 2). Their involvement in brassinosteroid signaling has not yet been studied. Moreover, FRK1 is also called SENESCENCE-INDUCED RLK (SIRK) since the gene is highly expressed during senescence (cf. TAIR homepage), when massive cell wall degradation occurs. 

Furthermore, the MALL-LRR-RLK CAMEL (CANALIZATION-RELATED AUXIN-REGULATED MALL-LRR-RLK, At1g05700) with three LRR repeats interacts and phosphorylates PIN-FORMED (PIN) auxin transporters together with CANAR (CANALIZATION-RELATED RLK). The CAMEL-CANAR receptor complex mediates PIN1 subcellular trafficking and localization and thus coordinates cell polarization during auxin canalization [213]. Finally, He et al. [214] showed that the MALL-LRR-RLK SALT INDUCED MALECTIN-LIKE DOMAIN-CONTAINING PROTEIN1 (SIMP1) interacts with, phosphorylates and thus stabilizes the putative proteasome maturation factor UMP1A (At1g67250) and promotes degradation of aberrant unfolded/misfolded proteins to mitigate salt-stress-induced ER stress, leading to enhanced salt tolerance in plants. Therefore, the SIMP1–UMP1A module is a regulator of plant salt tolerance [214]. Interestingly, the *sif1sif2* double knock-out line also shows improved salt tolerance [211] and the rice CARBOHYDRATE-BINDING MALECTIN-LIKE PROTEIN OsCBM1, which contains only a MALL domain, contributes to drought-stress tolerance by participating in NADPH oxidase-mediated ROS production [215]. These examples demonstrate that the MALL-domain-containing proteins also play a role in abiotic stress responses. Finally, the MALL-LRR-RLKs ROOT HAIR SPECIFIC (RHS)6 and -16 (Figure 1D) control root hair development [216] (Figure 2).

Several of the mRNA levels of these MALL-LRR-RLKs (such as for IOS1, FRK1 and SIF2) are upregulated in response to pathogen attacks, while those for MALL-RLKs are often downregulated [134]. This supports the idea that MALL-LRR-RLKs promote immune and other stress responses, while MALL-RLKs balance immune and other cellular processes.

### 5.3. A Lesson from Symbiosis

The Arabidopsis MALL-LRR-RLK IOS1 has a similar domain structure as the SYMBIOSIS RLK (LjSYMRK) from *Lotus japonicus*. LjSYMRK is required for both root nodule symbiosis and arbuscular mycorrhizal symbiosis [217,218], and the kinase is widely distributed in different plant species [219]. The symbiotic pathways from rhizobia and mycorrhizal fungi are induced by symbiosis-related LysM receptors upstream of SYMRK. The bacterial lipo-chitooligosaccharide is recognized by the plant NODULATION FACTOR RECEPTORs (NFRs) [220,221,222], whereas mycorrhizal fungi produce lipochitooligosaccharides similar to Nod factors, as well as short-chain chitin oligomers, implying commonalities in signaling during mycorrhizal and rhizobial associations [223]. Both pathways converge at SYMRK [224], and success of the symbiotic interactions is controlled by the abundance of SYMRK. Lower abundance of SYMRK due to the absence of symbiotic stimuli from rhizobia or mycorrhizal fungi restricts signaling towards beneficial interaction.

In the absence of the symbiotic signals, the MALL domain of SYMRK is released from the residual RLK, either by an extracellular protease or autocatalytic cleavage. Cleavage requires a conserved consensus motif “GDPC” downstream of the MALL domain. Hok et al. [209] and Antolín-Llovera et al. [218] describe that this motif is also present in the majority of the Arabidopsis MALL-LRR-RLKs (41 out of 60 proteins) after their MALL domains. Therefore, it is conceivable that this cleavage also occurs in other MALL-LRR-RLKs. This might be an important difference between MALL-LRR-RLKs and LRR-MAL-RLKs (cf. below): the latter kinases contain their MAL domains downstream of their LRR domains.

In *L. japonicus*, release of the MALL domain of LjSYMRK is associated with lower abundance and potentially rapid degradation of the residual RK [217]. Interestingly, chitin oligosaccharides induce immunity via similar LysM RLKs. CERK1 in rice has the highest homology to NFR1, and Zhang et al. [223] showed for rice that CERK1 is necessary for the establishment of the mycorrhizal interaction as well as for resistance to the rice blast fungus. This suggests that NFR1/CERK1 represents a common receptor in rice for chitooligosaccharide-based signals produced by mycorrhizal fungi, rhizobial bacteria (in legumes) and fungal pathogens. Mycorrhizal recognition has been conserved in multiple receptors across plant species, but additional diversification in certain plant species has defined other signals that this class of receptors can perceive [223]. In light of this regulatory circuit, potential cleavage of the MALL domains of MALL-LRR-RLKs might determine the stability of the remaining LRR-RLKs fragments and their potential interaction with other receptors or signaling compounds. As a consequence, either symbiotic or defense pathways can be activated, depending on the microbial signals and the receptor combinations of the plant. Therefore, it is important to test whether cleavage of the MALL domain, followed by internalisation and degradation of the remaining protein, is a general phenomenon of MALL-LRR-RLKs and whether this influences the perception of external signals and receptor interactions at the plasma membrane. Alterations in receptor abundance and interactions might be crucial for balancing symbiotic and defense responses. It might be possible that MALL-LRR-RLKs have a similar function as MALL-RLKs in balancing immune and growth responses.

### 5.4. MAL Domain-Containing RLKs

Phylogenetic analyses showed that only 13 proteins of the MAL/MALL domain-containing proteins are MAL-RLKs in Arabidopsis [129] (Figure 1). MAL-RLKs contain an N-teriminal LRR domain followed by the MAL domain before the transmembrane span. As outlined above, CORK1/IGP1 was identified as a cello-oligomer receptor (Figure 2). Cellotriose was the most effective cello-oligomer for the induction of intracellular signaling, where binding studies showed that besides cellotriose, cellopentaose also binds to the MAL domain of CORK1/IGP1 [6,7]. Martín-Dacal et al. [7] also showed that IGP2/3 (At1g56130) and IGP4 (At1g56140) are required for cytoplasmic Ca^2+^ elevation induced by mixed-linkage β-1,3/β-1,4 glucans. Isothermal titration calorimetry experiments could not show binding of mixed-linkage glucans to CORK1/IGP1 or IGP4, whereas cellotriose and cellopentaose bound to CORK1/IGP1. The authors hypothesized that the MAL-RLKs which do not bind directly to cello-oligomers, or mixed-linkage glucan-derived oligosaccharides potentially function as co-PRRs. Taken together, at least 3 of the 13 MAL-RLKs induce signaling in response to oligosaccharide breakdown products from cellulose and/or mixed-linkage glucans (Figure 1A and Figure 2).

The function of the other MAL-RLKs was investigated in different contexts (Figure 2). NEMATODE-INDUCED LRR-RLK 2 (NILR2, At1g53430) participates in the induction of innate immune responses to parasitic nematodes [225]. The LysM RK1-INTERACTING KINASE1 (LIK1, At3g14840) is a CERK1-interacting kinase, which regulates plant immune responses [226]. These examples link pathogen-induced immune responses to a member of the MAL-RLK family. LEUCINE-RICH REPEAT RECEPTOR-LIKE KINASE WITH EXTRACELLULAR MALECTIN-LIKE DOMAIN 1 (LMK1, At1g07650) is involved in cell death responses in Arabidopsis leaves, and the response is coupled to carbon/nitrogen-nutrient signaling pathways [227]. The RECEPTOR-LIKE KINASE IN FLOWERS1 (RKF1) and at least one of its paralogues RKF-LIKE (RKFL)1-3 are involved in early stages of the dialogue between pollen and pistil during egg fertilization [228]. It would be interesting to investigate whether cell wall rearrangements during pollen tube growth and crosstalk with the pistil activates signaling processes which are comparable to CWI signaling. Tissue-specific expression profiles demonstrate that many MAL- and MALL-RLK genes are highly expressed in growing pollen tubes and the female tissues during fertilization (e.g., TAIR homepage) besides expression in root or leaf tissues. Moreover, in angiosperm pollen tubes, the acto-myosin system controls secretory vesicle flow to the apex for polarized growth. Recent data on membrane trafficking suggest a role of microtubules in fine delivery and repositioning of vesicles to sustain pollen tube growth [229]. The role of polysaccharide biosynthesis, endomembrane vesicle flow, deposition and re-arrangement of cell wall material during pollen tube growth, pollen tube/pistil interaction as well as the pollen journey to the ovule have been described in detail by Onelli et al. [229]. They also emphasize the crosstalk between the male gametophyte and molecules of the pistil-secreted extracellular matrix which support, attract and guide the pollen tubes. All of these processes are tightly associated with massive alterations in the cell wall architecture, which are induced by cell internal or developmental signals. The vast majority of the regulatory processes described for MAL-RLKs requires secretion via endomembrane vesible flow. It appears that reversible phosphorylation events fine-tunes the secretory machinery via signals from MAL-RLKs. Finally, except for CORK1/IGP1, IGP2/3 and IGP4, the role of the MAL domain for the activation of the RLKs has not yet been investigated although all LRR-MAL-RLKs share high sequence similarities (Figure 1A). 

## 6. Mechanosensors in Monitoring CWI

### 6.1. Ideas from Fungi

A CWI pathway is highly conserved in the fungal kingdom and has been extensively investigated in yeast [230]. Although plant and fungal cell walls differ, there might be similarities in the perception and signaling. In yeast, cell wall damage is sensed at the plasma membrane through the cell-surface sensors Wsc1, -2, and -3, Mid2 and Mtl1 [231]. The receptors function as mechanosensors of cell wall stress and induce wall remodeling [232]. The sensors are activated by cell wall perturbations, perhaps by direct contact with the cell wall. Receptor activation stimulates nucleotide exchange on the small GTPase Rho1 and activation of Protein Kinase C1 (cf. [230,233]). The main role of activated Protein Kinase C1 is to trigger the MAPK module and the activation of downstream transcription factors which induce adaptive responses. The transcription factors ScRlm1 and the ScSwi4/ScSwi6 complex which induce the expression of genes related to cell wall biogenesis are well investigated [234,235].

The Wsc-family CWI sensors monitor stress-induced rearragments around the attachment sites of plasma membrane and cell wall regions. These sensors contain four domains: a cytoplasmic tail for signaling, a transmembrane domain, followed by a highly O-mannosylated serine-threonine region in the apoplast and finally a cysteine-rich domain which interacts with glucans and/or cell wall proteins [233]. O-mannosylation of their extracellular domains is required for function [235]. The sensor system uses the transmembrane domain as one anchor and the extracellular cysteine-rich domain in the cell wall as the other anchor. Disturbances in either the cell wall or stretching of the membrane could be detected as a force tilting and stretching the serine/threonine rich domain which connects the cysteine-rich domain with the transmembrane domain. This force results in a conformational change within the cytoplasmic tail of the Wsc receptor and activates the phosphorylation cascade (cf. details and models in [233]).

Besides mechanoperception, the yeast system also allows the perception of osmotic changes which alter the plasma membrane–cell wall connection [233]. It could therefore provide a model for plants. Fungal membrane RLKs that play a role in pH control, mechano-sensing and perception of apoplastic signals such as ROS may have equivalents in plants for CWI sensing. 

### 6.2. Mechanosensing in Plants

Besides the cell-surface sensors Wsc1, -2, and -3, Mid2 and Mtl1 [231], the yeast tugor pressure sensor SYNTHETIC LETHAL OF N-END RULE1 (SLN1) may be of relevance for CWI signaling in plants, since the yeast *sln1* mutant can be complemented with the ARABIDOPSIS HISTIDINE KINASE1 AHK1 ([236] and ref. therein). AHK1 is an osmosensor histidine kinase that responds to water stress. AHK1 and the yeast SNL1 might have similar osmo-sensing functions in their plasma membranes [50,237,238,239], and this similarity can be extended to the other three Arabidopsis histidine kinases AHK2-4. They are part of the two-component systems composed of hybrid histidine kinases, histidine-containing phosphotransfer domain proteins and response regulators that are biochemically linked by histidine-to-aspartate phosphorelay. In plants, they also play a role in cytokinin and ethylene perception and signalling besides osmosensing.

Mechanosensing proteins in plants were identified through the isolation and characterization of mutants. Toja et al. [240] established a screen which is based on unusual root mechanical behavior. Burri et al. [241] established a microelectromechanical systems-based force sensor which allows mimicking the pollen tube’s journey from stigma to ovary in vitro. Both approaches may help to identify mutants which perceive alterations in the cell wall or at the plasma membrane junction, although in different organs.

At least three types of channels participate in mechanosensing and the conversion of the physical into chemical signals via Ca^2+^ channel activities in plants: the mechanosensitive Ca^2+^ channels MECHANOSENSITIVE CHANNEL OF SMALL CONDUCTANCE (MCA), REDUCED HYPEROSDMOLALITY-INDUCED Ca^2+^ INCREASE (OSCA) and PIEZO [242,243]. These channels are activated by membrane tension and transfer information from the apoplast to the cytoplasm. Furthermore, at least two of the ten members of the MscS family participate in mechanosensing and perception of impairments to the cell wall/plasma membrane junction in Arabidopsis.

#### 6.2.1. MCA Proteins Are Mechanical Stress Sensors

The plasma membrane localized MCA proteins are found exclusively in land plants where the roots are exposed to new environmenal forces during the penetration through the soil. Thus, the presence of MCA proteins in land plants might be related to the specific selective pressure, which cannot rely on water buoyancy to support themselves and hence developed posture control mechanisms to maintain an erect habit [242,244]. MCA1 as well as its paralog MCA2 are involved in mechanical stress-induced Ca^2+^ influx in Arabidopsis. MCA channels are organized as a homotetramer with a small transmembrane region forming the pore and a larger cytoplasmic region which contain an EF hand-like motif, a coiled-coil motif and a cysteine-rich region. The latter region contains PLAC8 or DUF614 motifs which are crucial for regulating the pore (cf. [242], and ref. therein, [245]). Thus, the activity of MCA channels can be regulated by intracellular Ca^2+^ and protein interactions [246]. The EF hand-like region is necessary and sufficient for Ca^2+^ uptake, and the coiled-coil motif regulates MCA1 negatively and MCA2 positively. In addition, Yamanaka et al. [247] showed that MCA1 and MCA2 have distinct and overlapping roles in Arabidopsis. Yoshimura et al. [248] showed that MCA2 is directly activated by membrane tension and voltage. MCA1 and MCA2 are also involved in the perception of gravity signals and may be responsible for resistance to hypergravity [249,250]. They also mediate cold-induced cytoplasmic Ca^2+^ elevation and cold tolerance in Arabidopsis [251], and at least MCA2 is involved in touch-induced root responses in Arabidopsis [252,253]. Expression of Arabidopsis MCA1 enhanced mechanosensitive channel activity in the *Xenopus laevis* oocyte plasma membrane [254]. Furthermore, MCA1 participates in root penetration in hard agar media [247,253]. Okamoto et al. [255] showed that root growth inhibition in response to mechanical stress involves MCA1 and BAK1, as well as ethylene–mediated microtubule reorganization. The MCA1-BAK1 crosstalk suggests that MCAs are likely imbedded into a broader signaling network.

#### 6.2.2. OSCA Channels

The OSCA channels belong to the largest family of mechanosensors in fungi, animals and plants, with 15 genes in Arabidopsis. Yuan et al. [256] showed that OSCA1 mediates osmotic Ca^2+^ signalling in guard and root cells and is involved in transpiration regulation and root growth in response to osmotic stress. Procko et al. [257] suggested an involvement of the OSCA channels in the touch response of the hair of the Venus flytrap (*Dionaea muscipula*). Rice contains eleven OSCAs and multiple members of the OsOSCA family have redundant functions in osmotic sensing and stress adaption [258,259]. Maity et al. [260] analysed the structure of the rice OSCA1.2 and elucidated the mechanical basis of potential membrane hyperosmolality gating. The structure revealed a dimer; each subunit consists of 11 transmembrane helices and a cytosolic domain with homology to RNA recognition proteins. The transmembrane domain is structurally related to Ca^2+^-dependent ion channels and lipid scramblases, while the cytosolic domain possesses helical arms that are arranged parallel to the plasma membrane. These helical arms are well positioned to potentially sense lateral tension on the inner leaflet of the lipid bilayer caused by changes in turgor pressure.

#### 6.2.3. The MALL-RLK BUPS1 Participates in Mechanoperception

Recently, Zhou et al. [261] showed that Arabidopsis pollen tubes sense and/or respond to mechanical changes via BUPS1 while emerging from compressing female tissues. BUPS1-defective pollen tubes fail to maintain cell integrity after emergence from these tissues. The mechano-transduction function of BUPS1 was established by using a microfluidic channel device mimicking the mechanical features of the in vivo growth path. BUPS1-based mechano-transduction activates the ROP1 GTPase to promote exocytosis that facilitates secretion of BUPS1’s ligands for mechanical signal amplification and cell wall rigidification in pollen tubes. Therefore, a MALL-RLK is also involved in mechano-transduction in cells which copes with the physical challenges during invasive or penetrative growth [261].

#### 6.2.4. PIEZO Ion Channels Operate at Vacuolar Membranes

In mammals, PIEZO1 and PIEZO2 are mechanosensitive ion channels involved in touch perception or regulating the volume of red blood cells (cf. [262]). They convert mechanical forces into cytosolic Ca^2+^ signals and activate Ca^2+^-dependent downstream responses. With over 2000 amino acids that span the cell membrane dozens of times, the unusually large molecules do not share any known structural similarity with all other known channels involved in mechanosensing (cf. [262]). The mammalian PIEZO1 proteins form trimers and are shaped like a propeller with three blades organized around a central pore. When the channel is closed, the three blades curve out of the plane of the pore domain. This physically bows the membrane, creating the dome. When the tension on the membrane increases, the dome flattens, and the blades straighten, and this could supply the energy needed to open the channel ([262] and original refs. therein).

In plants, a PIEZO chanel was originally described to be involved in the immune response to viruses [263] before its involvement in mechanosensing in roots [243,264]. *PIEZO* promoter::*GUS* gene fusions were mainly expressed in the tip of the root cap in Arabidopsis [264]. Mousavi et al. [243] showed that a chimeric construct with the C-terminal part of AtPIEZO1 harboring the transmembrane pore and the N-terminal part of the mouse PIEZO1 accumulates in the plasma membrane, and the fusion protein is active in mammalian cells.

More recently, Radin et al. [265] investigated PIEZO function in tip-growing cells in the moss *Physcomitrium patens* and Arabidopsis. The *P. patens* PIEZO1 and PIEZO2 redundantly contributed to the normal growth, size, and cytoplasmic Ca^2+^ oscillations of caulonemal cells, and both proteins localized to vacuolar membranes. The moss PIEZO homologs promote increased complexity of vacuolar membranes through tubulation, internalization, and/or fission. Arabidopsis PIEZO1 also localized to the tonoplast in the tips of pollen tubes and is required for vacuole tubulation. Radin et al. [265] propose that in plant cells the tonoplast has more freedom of movement than the plasma membrane, making it a more effective location for mechanosensory proteins. This clearly raises questions about the crosstalks between the perception systems in different membranes, and whether common signaling compounds in the cytoplasm coordinate the responses.

#### 6.2.5. MscS

The MscS family of ion channels is involved in mechanosensing and has 10 members in Arabidopsis. Two MscS-Like (MSL) proteins, MSL9 and MSL10, are located in the plasma membrane of root cells [266]. MSL1 is localized to the inner mitochondrial membrane, where it is required for oxidative stress responses [267]. Charge–charge interactions modulate the mechanosensitive channel function at the inner mitochondrial membrane [267], and this might be linked to extracellular events as investigated by Dünser et al. [268]. They showed that apoplast acidification/cell wall loosening sensed by FER and LRR-extensins controls vacuolar expansion during cell elongation. The cytosol showed relatively minor volume expansion during epidermal elongation. Arabidopsis *fer* loss-of-function mutant vacuoles were markedly less affected by extracellular constraints. These studies demonstrate that plasma-membrane-localized CWI sensors crosstalk with sensors in mitochondrial membranes.

## 7. The FASCILIN-LIKE ARABINOGALACTAN PROTEIN4 (FLA4)-FEI Pathway

Several independent screens for mutants that respond to environmental signals for adjustments of the cell wall structure [269,270] identified FEI1 and FEI2 (for Chinese “fat”), plasma-membrane-localized co-receptors with LRR motifs involved in cell wall elongation and the extracellular glycoprotein FLA4. FLA4 was also identified in a salt stress tolerance screen and named SALT OVERLY SENSITIVE5 (SOS5, summarized in [270]), although the link of the FLA4-FEI pathways to salt tolerance is not clear. The FLA4/FEI control unit is linked to ethylene biosynthesis and its central enzyme AMINOCYCLOPROPANE 1-CARBOXYIC ACID (ACC) SYNTHASE. The ACC synthase is inhibited by binding to FEI1/FEI2, and binding requires dimerization of FEI1 and FEI2 and the activation of the kinase domain at the cytoplasmic site of the plasma membrane. This results in lower ACC production, less ethylene biosynthesis and reduced inhibition of cellulose synthesis. To activate the process, FLA4 in the apoplast must be glycosylated which is mediated by the arabinogalactan-protein-specific GALACTOSYLTRANSFERASE2 and -5 (GALT2/5). Quintuple mutant *galt2/galt5/sos5/fei1/fei2* analyses showed that the genes act in a single, linear genetic pathway [269]. FEI1/2 and MCA1 are also connected to THE1, whereas THE1 was supposed to act upstream of FEI2 (cf. [134,270]). Both loci, *THE1* and *FEI2*, antagonized PTI mediated by the PAMP peptides PEP1 and PEP3, suggesting that the pathway participates in balancing growth and PTI. Furthermore, the LRR-RK MALE DISCOVERER 1-INTERACTING RECEPTOR-LIKE KINASE 2 (MIK2) (At4g08850) connects CWI sensing, root growth and response to abiotic and biotic stresses [271], probably via FLA4/FEI and MIK2 crosstalk. Whether and how tight CWI signaling by the FLA4/FEI pathway is connected to other perception systems, needs to be investigated in detail. 

## 8. eATP-Induced Signaling Occurs during Massive Cell Wall Alterations

Extracellular adenosine 5′-triphosphate (eATP) can be considered as an inter-kingdom signaling molecule [272] and participates in the information transfer between cells from different organisms and systemic signal propagation within the plant [273,274]. Although eATP has long been discussed as a signaling molecule in animals [275,276,277,278] and plants [279,280], it is not yet clear how ATP is transferred from the cytoplasm to the extracellular space in response to specific signals. It can be released into the apoplast by (i) channels, (ii) transporters, and/or (iii) exocytosis [276]. In addition, wounding results in ATP release from the cytoplasm [281]. More specifically, the p-glycoprotein (PGP1) belonging to the ATP-binding cassette (ABC) transporters [282] and the plasma membrane-localized nucleotide transporter PM-ANT1 [283] have been identified in ATP export. 

eATP might also be involved in CWI responses. Lim et al. [284] postulated that eATP induces stress and growth responses (cf. also [285,286]). Feng et al. [287] describe the involvement of eATP in plant cell death. The eATP level is controlled by hydrolytic enzymes called apyrases (APYs) [285]. In Arabidopsis, APY1 and APY2 are located in the membranes of the Golgi apparatus and control the eATP level by modulating the luminal concentration of ATP in the secretory vesicles (cf. [274,288,289,290]. Suppression of *APY1* and *APY2* expression causes dwarfism, impaired polar auxin transport and eATP over-accumulation [274,291,292,293,294]. Furthermore, ecto-APYs control the eATP concentrations in the apoplast and might contribute to the growth/defense balance. In beneficial and pathogenic interactions, eATP hydrolyzing enzymes can be of plant or microbial origin.

eATP binds and thus activates the P2K1 receptor [279,280] [the L-type lectin RLKI.9 (LecRK-I.9); previous name: DOes not Respond to Nucleotides (DORN1)] which in turn activates Ca^2+^ channels. The P2K1 receptor binds eATP with its extracellular ATP-binding lectin domain and contains an intracellular kinase domain. *P2K1* is highly expressed during developmental and growth phases and several auxin-regulated processes [295] but also during stresses when massive cell wall rearrangements occur [274,275,281,296,297,298,299,300,301,302].

## 9. Extracellular Pyridine Nucleotides Inform Neighboring Cells

Similar to ATP, NAD(P)^+^ can be released into the apoplastic space. Besides activation of numerous immune responses through Ca^2+^- and SA-mediated pathways, the metabolite plays a crucial role in systemic acquired resistance (SAR) [303]. The lectin RK (LecRK), LecRK-VI.2, is a potential receptor for extracellular NAD^+^ (eNAD^+^) and NAD^+^ phosphate (eNADP^+^). LecRK-VI.2 constitutively associates with BAK1 in vivo, and the kinase activities of LecR-VI.2 and BAK1 are indispensable to their function in SAR [303]. Pathogen infection or cell death causes leakage of intracellular NAD(P) into the extracellular space [304]. Besides defense gene activation, the metabolite also controls processes required for cell wall repair (cf. [272,305,306]).

## 10. Crosstalk and Downstream Signaling

All described perception systems monitoring alterations in the apoplast crosstalk with each other either by protein/protein interactions or common signaling components. Elucidating the interaction network will probably be a major task for future investigations. Crosstalk to the PAMP-triggered immune systems are often linked to CWI perception via common signaling components such as BAK1.

The vast majority of studies on CWI signaling focuses on immune responses. This includes the activation of the MAPK3/6 module by phosphorylation and of transcription factors stimulating defense gene expression. Signaling via reversible phosphorylation is one of the fastest responses to apoplastic stimuli, and more and more target proteins besides MAPKs were identified. Nissen et al. [187] proposed that CrRLK1L interaction with GPI-AP and GEFs activate ROPs which induce NADPH oxidase-dependent ROS production to control the balance between cell-wall-loosening hydroxyl radicals and cell wall-rigidifying H_2_O_2_. RESPIRATORY BURST OXIDASE HOMOLOGUE D (RBOHD) is reversibly phosphorylated and ubiquitinated in response to DAMPs and PAMPs. The conformational changes in N-terminal EF-hand motifs of RBOHD allow phosphorylation by various kinases [307,308,309]. Tseng et al. [6] showed that cellotriose application results in rapid RBOHD phosphorylation in a CORK1/IGP1-dependent manner, indicating that cello-oligomers control ROS production in Arabidopsis. The MALL-LRR-RLK SIMP1 interacts with, phosphorylates and thus stabilizes the proteasome maturation factor UMP1A to promote degradation of misfolded proteins under salt stress [214]. CAMEL interacts and phosphorylates PIN transporters and participates in PIN1 subcellular trafficking and localization [213]. Finally, Gandhi et al. [310] analysed phosphoproteome data after cellotriose application to Arabidopsis roots and identified proteins involved in cellulose biosynthesis, the cellulose synthase complex formation and its trafficking from the ER to the plasma membrane. Control of membrane trafficking to the plasma membrane is important for cell wall repair, but also release of repair/defense compounds into the apoplast, and a number of proteins involved in the vesicle flow are rapidly activated by phosphorylation [310]. Likewise, BUPS1-based mechano-transduction activated the ROP1 GTPase to promote exocytosis that facilitates secretion of BUPS1’s ligands for mechanical signal amplification and cell wall rigidification in pollen tubes [261]. Membrane trafficking is apparently a major target of CWI signaling. Unraveling the crosstalk between DAMP-/PAMP-triggered signaling and developmental programs requires a deeper understanding of changes in the phosphoproteome patterns.

pH changes in the apoplast have major impact on signal perception. Auxin triggers apoplastic acidification by activating plasma membrane P-type H^+^-ATPases (AHAs) along with cell wall relaxation [311]. The apoplastic pH also changes during plant/microbe interactions and functions as an integrator of signaling in roots [312,313]. This has profound influence on protein/protein interactions, their enzyme activities and signaling. RALFs provide an example of how plants use apoplastic pH changes to induce appropriate answers of the cell. To what extend the observed perception and signaling processes are dependent on apoplastic pHs might be important for future studies.

## Figures and Tables

**Table 1:** Main plant cell wall-derived oligosaccharides from cell wall polysaccharides with elicitor function in immunity and wall growth/repair.

Oligosaccharides	Origin	Receptor	References
cello-oligomer, n 2–7 (β-1,4-glucans)	cellulose	CORK1/IGP1	[6,7,8,9,10,11,12]
β-1,3-glucans, non-branched	callose	CERK1 (for short-chain β-1,3-glucans), LYK4, LYK5	[13,14]
α-1,4-oligogalacturonides (OGs)	pectin	WAKs	[12,13,15,16]
arabinoxylan	xylan		[17]
mixed-linkage β-1,3/1,4 glucan oligosaccharides	mixed-linkage glucan polysaccharides	IGP2/3, IGP4	[7,18,19,20]
xyloglucan oligosaccharides	xyloglucan		[21]
mannan oligosaccharides, n = 2–6	mannan polysaccharides		[22]

## Data Availability

Not applicable.
